# Validation of the Italian HeartQoL: a short health-related quality of life questionnaire for patients with ischemic heart disease

**DOI:** 10.1007/s11739-021-02780-2

**Published:** 2021-06-10

**Authors:** Francesco Fattirolli, Alessia Argirò, Maria Elisabetta Angelino, Gianluigi Balestroni, Francesco Giallauria, Daniela Miani, Carlo Vigorito, Lucrezia Piccioli, Franco Tarro Genta, Stefan Höfer, Niccolò Marchionni, Neil Oldridge

**Affiliations:** 1 Department of Experimental and Clinical Medicine, University of Florence and Cardiothoracovascular Department Azienda Ospedaliero-Universitaria Careggi, Largo Brambilla 3, 50141 Firenze, Italy; 2 Psychology Unit, Istituti Clinici Scientifici Maugeri, Turin, Italy; 3 Psychology Unit, Istituti Clinici Scientifici Maugeri, Veruno, Italy; 4 Department of Translational Medical Sciences, Federico II University of Naples, Naples, Italy; 5 Cardiology Unit, University Hospital S. Maria Della Misericordia, Udine, Italy; 6 Department of Cardiology Istituti Clinici Scientifici Maugeri, Turin, Italy; 7 Department of Medical Psychology, Medical University of Innsbruck, Innsbruck, Austria; 8 College of Health Sciences, University of Wisconsin-Milwaukee, Milwaukee, WI USA

**Keywords:** Health-related quality of life, Italian HeartQoL questionnaire, Angina pectoris, Myocardial infarction, Ischemic heart failure, Validation

## Abstract

The psychometric properties of the core disease-specific 14-item Italian HeartQoL health-related quality of life questionnaire have been evaluated in this study. The Italian version of the HeartQoL, the MacNew questionnaire, and the Hospital Anxiety and Depression Scale were completed by 472 patients (angina, *N* = 183; myocardial infarction, *N* = 167; or ischemic heart failure, *N* = 122) who were recruited in five Italian centers (Florence, Veruno, Turin, Udine, and Naples) between 2015 and 2017. Patients with myocardial infarction reported significantly higher HeartQoL scores than patients with angina or ischemic heart failure. Floor and ceiling effects were always minor on the HeartQoL global scale and physical subscale with moderate ceiling effects on the emotional subscale in the total group and in patients with myocardial infarction. The bifactorial structure of the original HeartQoL questionnaire was confirmed with strong physical, emotional, and global scale H coefficients (> 0.50). The HeartQoL scales demonstrated optimal internal consistency (Cronbach’s alpha > 0.84). Convergent and divergent validity were confirmed. Discriminative validity was not confirmed for age, largely confirmed for sex, and fully confirmed for anxiety, depression, and distress. The Italian HeartQoL questionnaire demonstrated adequate key psychometric attributes of internal consistency reliability and validity in Italian-speaking patients with ischemic heart disease.

## Background

Critical steps in improving the quality of health care include evidence-based, patient-centered, and systems-oriented care [1]. Incorporating this approach has changed medicine in all areas, from everyday practice, where shared decision making has become essential and recommended by guidelines [2] to clinical trials where patients are involved in Steering Committees [3]. Major treatment goals for patients with cardiovascular disease include a focus on health status which includes symptom burden, functional limitations, and health-related quality of life (HRQL) [4, 5]. Health status typically is evaluated with patient-reported outcome questionnaires through which patients may express feelings about their health condition without adulteration or interpretation by clinicians [6].

Both generic and disease-specific patient-reported outcome questionnaires can be used to evaluate and compare the health status. Generic instruments, such as the SF-36 Health Survey (SF-36) [7, 8], are designed for use in healthy populations, different disease populations as well as between healthy populations and populations with various diseases. On the other hand, disease-specific instruments, such as the Seattle Angina Questionnaire [9] and the Minnesota Living with Heart Failure questionnaire [10] provide more relevant disease-specific information than generic instruments. However, there is a need for core disease-specific questionnaires which permit between-diagnosis comparisons within a given disease with a single questionnaire, for example, the MacNew [11] and, more recently, the HeartQoL [12, 13] in patients with ischemic heart disease (IHD).

The HeartQoL questionnaire was developed with the objective of assessing HRQL using a single instrument in patients with angina pectoris (AP), myocardial infarction (MI), or ischemic heart failure (IHF). The HeartQoL Project was conducted between 2002 and 2011 in European and English speaking regions in two phases: first, a cross-sectional survey to identify items for questionnaire inclusion on the basis of three previously validated instruments [12], the Seattle Angina Questionnaire, [9] the MacNew Heart Disease Health-related Quality of Life Questionnaire (MacNew) [11], and the Minnesota Living with Heart Failure questionnaire [10], and a second phase to evaluate the HeartQoL questionnaire’s psychometric properties [13]. The result was a bifactorial, 14-item questionnaire that was reliable, valid, and responsive in patients with AP, MI, or IHF [13]. These results have been recently confirmed in German [14] and Chinese [15] and also in other clinical contexts, such as atrial fibrillation [16], implantable cardioverter defibrillator recipients [17], and valve surgery [18].

To optimize their use as both research and clinical tools, patient-reported HRQL questionnaires need to meet basic attributes such as reliability and validity [19]. Reliability is considered the degree to which a questionnaire is free from random error with internal consistency reliability the intercorrelations of items at one point in time. Validity is the degree to which the instrument really measures what it purports to measure and includes convergent validity (the degree to which two theoretically related measures are in fact related), divergent validity (the degree to which two dissimilar measures can be differentiated) and discriminative validity (does the questionnaire adequately discriminate between groups that should or should not differ). The purpose of this study was to examine the factor structure, reliability, convergent, divergent, and discriminative validity of the Italian version of the HeartQoL questionnaire in patients with AP, MI, or IHF.

## Methods

### Patients

Patients diagnosed with IHD [*n* = 472] were recruited at five sites in Italy (Florence, Veruno, Turin, Udine, and Naples) from 2015 to 2017. The eligibility criteria were extracted from the original cross-sectional survey phase of the HeartQoL Project [12] and included:

(a) currently being treated for AP [20] with an objective measure of IHD (e.g., previous MI, exercise testing, echocardiogram, nuclear cardiac imaging, or coronary angiography); or

(b) had experienced a documented MI between one to six months previously; or

(c) currently being treated for IHF with evidence of left ventricular dysfunction (ejection fraction ≤ 40% by invasive or non-invasive testing) and an objective measure of IHD (e.g., exercise testing, echocardiogram, nuclear cardiac imaging, or coronary angiography).

The patients were ≥ 18 years, able to complete the self-administered battery of HRQL questionnaires in Italian, without hospitalization in the last 6 weeks, without serious psychiatric disorder as well as no current substance abuse as identified by the referring physician. The Ethics Committee in each study site approved the project and informed consent was obtained from all patients.

## Patient-reported questionnaires

### Sociodemographic and clinical variables

Demographic characteristics of age, sex, family status, education, employment, and cardiovascular risk factors (diabetes, hypercholesterolemia, hypertension, physical inactivity, and smoking) were self-reported by patients.

### HeartQoL

The Italian version of the disease-specific HeartQoL was used in the HeartQoL Project [12] and in this study to measure HRQL. The HeartQoL is a bi-dimensional instrument with a 10-item physical subscale and a 4-item emotional subscale that, when combined, provides the 14-item global scale [12]. The responders answer the question of how much they are bothered by each of the 14 HeartQoL questionnaire items on a 4-point scale ranging from “bothered a lot” (= 0) to “not bothered” (= 3) with higher scores indicating better HRQL. Both the global scale and the subscales were calculated as the mean of the scored items.

### Hospital anxiety and depression scale (HADS)

The HADS is a 14-item self-assessment questionnaire including an anxiety and a depression subscale that together generate a common score for general distress; the Italian version was used in this study [21, 22]. The items are answered on a scale ranging from 0 to 3 with higher scores representing higher levels of anxiety or depression. A subscale cutoff score ≥ 8 identifies patients with symptoms of depression or anxiety [22] and a global cutoff score ≥ 12 indicates “general” distress.

### MacNew

The MacNew Questionnaire is designed to assess patient’s feelings about how IHD affects daily functioning [11]. It contains 27 items with a global HRQL score and physical limitation, emotional and social function subscales, scored from 1 (low HRQL) to 7 (high HRQL) [23, 24]. Using forward–backward translation, the MacNew questionnaire had been translated into Italian and validated as part of the HeartQoL Project [25].

## Statistical analyses

Means and standard deviation (SD) are reported for continuous variables and frequencies for categorical variables. Categorical sociodemographic and clinical variables were analyzed with Pearson’s *Chi*-square test while continuous variables and scale means were examined with analyses of co-variance (ANCOVA) with post hoc Bonferroni correction adjusting for age, sex, risk factors, and disease severity within diagnosis. A two-sided *p* value of < 0.05 was applied for statistical significance. The data were analyzed using IBM SPSS Statistics 24 and STATA 14.

The evaluation of the psychometric properties of the Italian HeartQoL followed criteria recommended by the Scientific Advisory Committee [19]. Floor and ceiling effects of the HeartQoL are considered moderate when > 15% patients report the lowest score (= 0; floor) or the highest score (= 3; ceiling) [26]. A confirmatory Mokken scale analysis, using Loevinger’s coefficient *H* of ≥ 0.50 considered a “strong,” 0.49–0.40 a “moderate,” and 0.39–0.30 a “weak” scale [27], was performed to assess whether the established two-factor structure of HeartQoL [12] could be confirmed in the Italian HeartQoL. Internal consistency reliability was determined using Cronbach’s alpha with values of ≥ 0.70 acceptable for group and ≥ 0.90 for individual comparisons [28]. The strength of the correlations between HeartQoL and related (convergent) as well as unrelated (divergent) constructs was assessed with Pearson coefficients (*r* < 0.10 = no correlation, *r* = 0.10–0.29 = low correlation, *r* = 0.30–0.49 = moderate correlation, *r* ≥ 0.50 = high correlation) with Steiger’s test used for differences in correlations [29]. Discriminative validity was tested for using the “known-groups” approach [26] with paired t tests used to compare HeartQoL scores with two-level variables and one-way ANOVA used to compare three-level variables. The following discriminative validity hypotheses test whether significantly higher HeartQoL scores are reported:

1. by younger than by middle-age or older patients,
2. by male patients than by female patients,
3. by patients with MI than by patients with either AP or IHF,
4. by patients reporting higher MacNew HRQL scores (top 33% of actual scores) than by lower scores (bottom 33% of actual scores),
5. by patients without HADS anxiety (score < 8) than by patients with HADS anxiety (score ≥ 8)
6. by patients without HADS depression (score < 8) than by patients with HADS depression (score ≥ 8) and
7. by patients without HADS general distress (score < 12) than by patients with HADS general distress (score ≥ 12).

## Results

### Patient characteristics (Table 1)

**Table 1:** Patient characteristics: sociodemographic, risk factor, and intervention variables in patients with angina (AP), myocardial infarction (MI), or ischemic heart failure (IHF)

	Total cohort (*N* = 472)	AP (*N* = 183; 38.8%)	MI (*N* = 167; 35.4%)	IHF (*N* = 122; 25.8%)	*P* value
Age					
Years ± SD	65.4 ± 12.5	67.5 ± 10.9	63.1 ± 11.4	65.3 ± 14.5	0.004
Gender					
Female	120 (25.4%)	52 (28.4%)	36 (21.5%)	32 (26.2%)	ns
Male	352 (74.6%)	131 (72.6%)	131 (78.4%)	90 (73.8%)	
Marital Status (*N* = 463)					
Single	67 (14.5%)	26 (14.6%)	22 (13.3%)	19 (15.8%)	ns
Married/partnership	355 (76.7%)	134 (75.3%)	129 (78.2%)	92 (76.7%)	
Other	41 (8.8%)	18 (10.1%)	14 (8.5%)	9 (7.5%)	
Education (*N* = 460)					
< High school	239 (51.9%)	96 (53.3%)	82 (50%)	61 (52.6%)	ns
= High school	172 (37.4%)	68 (37.8%)	67 (40.9%)	37 (31.9%)	
> High school	49 (10.7%)	16 (8.9%)	15 (9.2%)	18 (15.5%)	
Employment (*N* = 461)					
Unemployed	45 (9.8%)	14 (7.82%)	16 (9.8%)	15 (12.6%)	ns
Retired	253 (54.9%)	113 (63.1%)	75 (46.0%)	65 (54.6%)	ns
Employed	163 (35.3%)	52 (29.1%)	72 (44.2%)	39 (32.8%)	0.017
Risk factors					
BMI	27.0 ± 4.7	27.2 ± 4.4	26.9 ± 4.4	26.9 ± 5.5	ns
Diabetes ^§^	113 (24.0%)	49 (26.9%)	33 (19.8%)	31 (25.6%)	ns
Hypercholesterolemia ^§^	229 (50.6%)	88 (50.6%)	95 (59.4%)	46 (38.7%)	0.003
Hypertension ^§^	249 (53.8%)	108 (60%)	91 (55.8%)	50 (41.7%)	0.006
Physical inactivity ^#^	213 (47.1%)	84 (48.35)	67 (41.4%)	62 (53.4%)	ns
Smoking	79 (17.2%)	28 (16%)	30 (18.1%)	21 (17.8%)	ns
Intervention					
PTCA	166 (35.5%)	49 (27.1%)	101 (60.5%)	16 (13.3%)	0.001
CABG	60 (12.8%)	32 (17.6%)	15 (9%)	13 (10.7%)	0.041
Valve surgery	19 (4.0%)	4 (2.2%)	4 (2.4%)	11 (9.1%)	0.005

*N* number of patients (sub-group *N* does not always = 472 due to missing data)

*p* value between diagnosis using *Chi*-square tests with categorical variables and ANCOVA with continuous variables (post hoc Bonferroni correction and scores adjusted for age, sex, and risk factors);

*BMI* body mass index, ^§^ Physician reported, ^#^ Patient-reported active on < 3 occasions per week, *PTCA* percutaneous transluminal coronary angioplasty, *CABG* coronary artery bypass grafting

A total of 472 patients with documented AP (*N* = 183, 38.8%), previous MI (*N* = 167, 35.4%) or IHF (*N* = 122, 25.8%) were eligible for the validation of the Italian HeartQoL Questionnaire. The mean age of the total cohort was 65.4 ± 12.5 years. The majority of patients (74.6%) were male, most were married (76.7%), 52% had less than high school education, and 54.9% were retired. Hypertension was the most prevalent risk factor (53.8%) followed by hypercholesterolemia (50.6%) and physical inactivity (47.1%) with differences in risk factors by diagnosis detailed in Table 1.

### Patient-reported questionnaire score distributions (Table 2)

**Table 2:** Scores (mean ± standard deviation) on HeartQoL, Hospital Anxiety, and Depression Scale (HADS) and MacNew and percentage HADS anxious, depressed, and distressed in the total cohort and in patients with angina (AP), myocardial infarction (MI), or ischemic heart failure (IHF)

	Total cohort (*N* = 472)	AP (*N* = 183; 38.8%)	MI (*N* = 167; 35.4%)	IHF (*N* = 122; 25.8%)	*P*-value
HeartQoL					
Global scale	2.0 ± 0.7	1.9 ± 0.7	2.3 ± 0.6	1.7 ± 0.8	< 0.001^a, b^
Physical subscale	1.9 ± 0.8	1.9 ± 0.8	2.2 ± 0.7	1.6 ± 0.9	< 0.001 ^a, b^
Emotional subscale	2.2 ± 0.8	2.1 ± 0.8	2.3 ± 0.7	2.0 ± 0.8	< 0.002^a, b^
MacNew					
Global scale	5.2 ± 1.1	5.1 ± 1.0	5.5 ± 1.0	4.9 ± 1.1	< 0.001^a, b^
Physical subscale	5.2 ± 1.2	5.1 ± 1.2	5.6 ± 1.0	4.9 ± 1.2	< 0.001^a, b^
Emotional subscale	5.1 ± 1.3	5.0 ± 1.1	5.3 ± 1.1	4.9 ± 1.2	< 0.002^a, b^
HADS					
Anxiety score	5.2 ± 3.9	5.5 ± 4.0	5.0 ± 4.1	5.2 ± 3.8	ns
Anxious (score, ≥ 8)	130 (27.1%)	47 (25.7%)	46 (27.5%)	35 (28.7%)	ns
Depression score	5.5 ± 4.2	5.7 ± 4.0	4.7 ± 4.2	6.3 ± 4.4	< 0.001^a^
Depressed (score, ≥ 8)	132 (27.9%)	57 (30.4%)	37 (22.2%)	39 (32.2%)	ns
General distress score	10.7 ± 7.4	11.2 ± 7.2	9,7 ± 7.6	11.5 ± 7.5	ns
Distressed (score, ≥ 12)	194 (41.1%)	79 (40.7%)	58 (29.9%)	57 (29.4%)	ns

*p* value between diagnosis using ANCOVA (post hoc Bonferroni correction and scores adjusted for age, sex, and risk factors)

^a^MI vs. HF

^b^MI vs. AP

*HeartQoL* Mean HeartQoL HRQL scores for the total cohort were 2.0 ± 0.7 on the global scale, 1.9 ± 0.8 on the physical subscale and 2.2 ± 0.8 on the emotional subscale with ANCOVA results demonstrating significantly higher global scale and both physical and emotional score differences in patients with MI than patients with either AP or IHF.

*MacNew* The mean MacNew global HRQL score was 5.2 ± 1.1, the mean physical subscale score was 5.2 ± 1.2 and the mean emotional subscale score was 5.1 ± 1.3 in the total group. Using ANCOVA, there were significant score differences between patients with MI and those with either AP or IHF on the global scale and both physical and emotional subscales with patients with MI reporting higher HRQL than patients with either AP or IHF.

*Hospital Anxiety and Depression Scale* For the total cohort, the mean HADS anxiety score was 5.2 ± 3.9 with anxiety scores ≥ 8 reported by 27.1% of the total cohort. The mean HADS depression score in the total cohort was 5.5 ± 4.2 with depression scores ≥ 8 reported by 27.9% of the total cohort. The mean HADS general distress score in the total cohort was 10.7 ± 7.4 with general distress scores ≥ 12 reported by 41.1% of the total cohort. Although patients with IHF reported significantly higher depression scores than patients with MI, the anxiety scores, the general distress scores and the proportions of patients with HADS anxiety or depression scores ≥ 8 or general distress scores ≥ 12 were not significantly different by diagnosis.

### Mokken scale factor structure (Table 3)

**Table 3:** Mokken scale analysis in in the total cohort for the HeartQoL items with Loevinger’s *Hi* (item) and *H* (scale) coefficients (strong, ≥ 0.50, moderate, 0.49 to 0.40)

Item	Global Hi (*N* = 433)	Physical Hi (*N* = 460)	Emotional Hi (*N* = 462)
1. Walk indoors on level ground?	0.57	0.64	
2. Garden. vacuum. or carry groceries?	0.62	0.70	
3. Climb a hill or a flight of stairs without stopping?	0.62	0.69	
4. Walk more than 100 yards at a brisk pace?	0.62	0.70	
5. Lift or move heavy objects?	0.62	0.68	
6. Feeling short of breath?	0.56	0.61	
7. Being physically restricted?	0.66	0.69	
8. Feeling tired. fatigued. low on energy?	0.65	0.67	
9. Not feeling relaxed and free of tension?	0.49		0.69
10. Feeling depressed?	0.47		0.70
11. Being frustrated?	0.55		0.63
12. Being worried?	0.53		0.72
13. Being limited in doing sports or exercise?	0.60	0.63	
14. Working around the house or yard?	0.61	0.67	
HeartQoL H	0.59	0.67	0.68

With Mokken analysis, the HeartQoL H coefficients were rated as “strong” on the global scale (0.59) and on both the physical (0.67) and the emotional (0.68) subscales confirming the original HeartQoL two-factor structure. Of the HeartQoL Hi coefficients, 85.8% of the items on the global scale (12 of 14 items; Hi range, 0.47–0.66) and 100% of the items on both the physical subscale (10 items; Hi range, 0.61 to 0.70) and the emotional subscale (4 items; Hi range, 0.63 to 0.72) were rated as “strong”.

### HeartQoL floor and ceiling effects (Table 4)

**Table 4:** Italian HeartQoL floor and ceiling effects and internal consistency reliability (Cronbach’s alpha) in the total cohort and in patients with angina (AP), myocardial infarction (MI), or ischemic heart failure (IHF)

HeartQoL	Total cohort (*N* = 472)	AP (*N* = 183; 38.8%)	MI (*N* = 167; 35.4%)	IHF (*N* = 122; 25.8%)
Global scale				
Floor effect	0.4%	1.0%	0.6%	1.0%
Ceiling effect	4.7%	3.8%	7%	2.50%
Cronbach’s alpha	0.94	0.94	0.93	0.94
Physical subscale				
Floor effect	0.6%	1.0%	1.2%	1.0%
Ceiling effect	7.4%	6.0%	10.8%	4.90%
Cronbach’s alpha	0.94	0.93	0.92	0.95
Emotional subscale				
Floor effect	1.7%	1.0%	0.6%	1.6%
Ceiling effect	16.5%	14.8%	22.2%	11.5%
Cronbach’s alpha	0.87	0.88	0.86	0.84

*N* number of patients;

Floor effect > 15% reporting poorest HRQL

Ceiling effect > 15% reporting highest HRQL

Floor effects on the HeartQoL global scale and both subscales were always ≤ 1.7% in the total group and in each diagnosis.

Ceiling effects on the HeartQoL global scale and the physical subscale were always ≤ 10.8% in the total group and in each diagnosis with moderate ceiling effects on the emotional subscale as high as 16.5% in the total group and 22.2% in patients with MI.

### Psychometric properties of the HeartQoL (Tables  2, 4, 5, & 6)

*Reliability (*Table 4) Internal consistency reliability was confirmed with Cronbach’s alpha ranging from 0.87 to 0.94 in the total cohort, from 0.88 to 0.94 in patients with AP, from 0.86 to 0.93 in patients with MI and from 0.84 to 0.95 in patients with IHF.

*Convergent validity *(Table 5) Convergent validity was confirmed in the total cohort and each diagnosis with correlations between the HeartQoL and MacNew physical subscales ranging from 0.71 to 0.75 and from 0.79 to 0.84 between the HeartQoL and MacNew emotional subscales. Correlations between dissimilar scales of both instruments were significantly lower according to Steiger’s test for comparing Pearson correlations confirming divergent validity.

**Table 5:** Convergent validity of the Italian HeartQoL subscales with the MacNew subscales in the total cohort and in patients with angina pectoris (AP), myocardial infarction (MI), or ischemic heart failure (IHF)

	HeartQoL		
	Physical subscale	Emotional subscale	*p*-value#
Total cohort (*N* = 472)			
MacNew Physical	**0.75**	0.70	0.05
MacNew Emotional	0.57	**0.81**	< 0.001
* p* value#	< 0.001	< 0.001	
AP (*N* = 183)			
MacNew Physical	**0.74**	0.71	0.427
MacNew Emotional	0.59	**0.81**	< 0.001
* p* value#	< 0.01	< 0.05	
MI (*N* = 167)			
MacNew Physical	**0.71**	0.72	0.094
MacNew Emotional	0.57	**0.84**	< 0.001
* p* value#	< 0.05	< 0.01	
IHF (*N* = 122)			
MacNew Physical	**0.73**	0.64	0.274
MacNew Emotional	0.51	**0.79**	< 0.001
* p* value#	< 0.01	< 0.01	

*N* number of patients;

Strong Pearson’s correlation coefficients between similar HeartQoL and MacNew subscales are bolded; all correlation coefficients, *p* < 0.01

^*#*^Steiger’s test for comparing Pearson’s correlations

*Discriminative validity *(Tables 2, 6) Discriminative validity for the HeartQoL was fully confirmed for diagnosis, MacNew global HRQL scores, HADS anxiety, depression, and general distress, largely confirmed for sex, and essentially not confirmed for age.

**Table 6:** Discriminative validity of the Italian HeartQoL in the total cohort and in patients with angina pectoris (AP), myocardial infarction (MI), or ischemic heart failure (IHF)

HeartQoL	Global scale	Physical subscale	Emotional subscale
Total cohort (N = 472)			
Age			
Young (*N* = 148)	2.08 [1.96–2.20]	2.1 [1.92–2.18]	2.17 [2.04–2.30]
Middle-age (*N* = 162)	2.01 [1.90–2.13]	1.95 [1.83–2.08]	2.16 [2.04–2.28]
Elderly (*N* = 151)	1.89 [1.78–2.01]	1.78 [1.65–1.91]	2.18 [2.06–2.30]
*p* value	ns	< .05; #	ns
Sex			
Female (*N* = 120)	2.09 [2.01–2.17]	2.02 [1.94–2.11]	2.25 [2.17–2.32]
Male (*N* = 351)	1.71 [1.58–1.85]	1.63 [1.49–1.77]	1.92 [1.78–2.09]
* p* value	< .001	< .001	< .001
MacNew—global score			
Top tertile (*N* = 154)	2.60 [2.54–2.63]	2.52 [2.45–2.60]	2.77 [2.73–2.81]
Bottom tertile (*N* = 157)	1.29 [1.20–1.39]	1.25 [1.14–1.36]	1.40 [1.29–1.52]
* p value*	< .001	< .001	< .001
HADS*			
Anxious (*N* = 128)	1.40 [1.28–1.53]	1.43 [1.30–1.57]	1.33 [1.20–1.46]
Not anxious (*N* = 343)	2.21 [2.15–2.28]	2.11 [2.03–2.19]	2.47 [2.41–2.53]
* p* value	< .001	< .001	< .001
Depressed (*N* = 131)	1.37 [1.25–1.49]	1.36 [1.22–1.49]	1.41 [1.27–1.54]
Not depressed (*N* = 337)	2.24 [2.18–2.31]	2.15 [2.07–2.23]	2.46 [2.41–2.52]
* p* value	< .001	< .001	< .001
Stressed (*N* = 194)	1.48 [1.39–1.58]	1.46 [1.35–2.58]	1.53 [1.43–1.64]
Not stressed (*N* = 227)	2.35 [2.29–2.41]	2.25 [2.17–2.34]	2.60 [2.56–2.65]
* p* value	< .001	< .001	< .001
AP (*N* = 183)			
*Age*			
Young (*N* = 41)	1.95 [1.71–2.20]	1.93 [1.67–2.20]	2.02 [1.73–2.30]
Middle age (*N* = 64)	1.97 [1.79–2.15]	1.92 [1.72–2.12]	2.10 [1.92–2.29]
Elderly (*N* = 74)	1.90 [1.73–2.07]	1.79 [1.60–1.97]	2.17 [1.99–2.35]
* p* value	ns	ns	ns
*Sex*			
Female (*N* = 52)	2.00 [1.88–2.13]	1.93 [1.79–2.07]	2.17 [2.04–2.31]
Male (*N* = 130)	1.74 [1.53–1.95]	1.68 [1.46–1.90]	1.90 [1.67–2.12]
* p* value	< .05	ns	< .05
*MacNew—global*			
Top tertile (*N* = 70)	2.60 [2.50–2.69]	2.52 [2.39–2.65]	2.78 [2.70–2.85]
Bottom tertile (*N* = 51)	1.33 [1.17–1.49]	1.30 [1.12–1.48]	1.42 [1.23–1.61]
* p value*	< .001	< .001	< .001
HADS*			
Anxious (*N* = 47)	1.32 [1.10–1.55]	1.38 [1.13–1.62]	1.20 [0.96–1.43]
Not anxious (*N* = 135)	2.14 [2.03–2.24]	2.03 [1.91–2.15]	2.41 [2.32–2.49]
* p* value	< .001	< .001	< .001
Depressed (*N* = 55)	1.35 [1.16–1.53]	1.33 [1.13–1.53]	1.40 [1.18–1.61]
Not depressed (*N* = 125)	2.19 [2.08–2.30]	2.10 [1.98–2.23]	2.41 [2.31–2.51]
* p* value	< .001	< .001	< .001
Stressed (*N* = 79)	1.45 [1.30–1.61]	1.44 [1.27–1.62]	1.48 [1.31–1.65]
Not stressed (*N* = 103)	2.29 [2.19–2.40]	2.18 [2.05–2.31]	2.57 [2.48–2.65]
* p* value	< .001	< .001	< .001
MI (*N* = 167)			
Age			
Young (*N* = 66)	2.31 [2.14–2.47]	2.29 [2.12–2.47]	2.34 [2.16–2.51]
Middle age (*N* = 59)	2.29 [2.14–2.44]	2.26 [2.10–2.42]	2.36 [2.18–2.53]
Elderly (*N* = 38)	2.13 [1.92–2.34]	2.06 [1.82–2.29]	2.31 [2.08–2.54]
* p* value	ns	ns	ns
Sex			
Female (*N* = 36)	2.33 [2.23–2.43]	2.31 [2.21–2.42]	2.36 [2.24–2.48]
Male (*N* = 131)	2.00 [1.77–2.22]	1.91 [1.66–2.16]	2.22 [1.98–2.45]
* p* value	< .005	< .001	ns
*MacNew—global*			
Top tertile (*N* = 77)	2.63 [2.56–2.70]	2.57 [2.47–2.67]	2.77 [2.71–2.84]
Bottom tertile (*N* = 34)	1.40 [1.22–1.58]	1.44 [1.23–1.64]	1.32 [1.08–1.55]
* p value*	< .001	< .001	< .001
HADS*			
Anxious (*N* = 46)	1.71 [1.52–1.89]	1.76 [1.56–1.96]	1.57 [1.36–1.78]
Not anxious (*N* = 121)	2.47 [2.38–2.55]	2.40 [2.30–2.51]	2.62 [2.54–2.70]
* p* value	< .001	< .001	< .001
Depressed (*N* = 37)	1.67 [1.44–1.90]	1.72 [1.47–1.96]	1.53 [1.25–1.81]
Not depressed (*N* = 130)	2.43 [2.34–2.51]	2.37 [2.27–2.47]	2.56 [2.48–2.64]
* p* value	< .001	< .05	< .001
Stressed (*N* = 58)	1.77 [1.60–1.93]	1.79 [1.61–1.98]	1.70 [1.50–1.90]
Not stressed (N = 109)	2.52 [2.44–2.60]	2.46 [2.36–2.56]	2.67 [2.60–2.73]
* p* value	< .001	< .001	< .001
IHF (*N* = 122)			
Age			
Young (*N* = 41)	1.85 [1.62–2.09]	1.77 [1.50–2.05]	2.04 [1.79–2.29]
Middle age (*N* = 39)	1.66 [1.40–1.92]	1.54 [1.25–1.82]	1.96 [1.68–2.25]
Elderly (*N* = 39)	1.66 [1.40–1.91]	1.49 [1.19–1.79]	2.08 [1.84–2.31]
* p* value	ns	ns	ns
Sex			
Female (*N* = 32)	1.87 [1.71–2.03]	1.93 [1.79–2.07]	2.17 [2.04–2.31]
Male (*N* = 90)	1.35 [1.11–1.59]	1.68 [1.46–1.90]	1.90 [1.67–2.12]
* p* value	< .001	ns	< .05
MacNew—global			
Top tertile (*N* = 43)	2.50 [2.32–2.69]	2.40 [2.13–2.66]	2.77 [2.68–2.86]
Bottom tertile (*N* = 26)	1.17 [1.01–1.33]	1.06 [0.87–1.26]	1.44 [1.24–1.63]
* p value*	< .001	< .001	< .001
HADS*			
Anxious (*N* = 35)	1.12 [0.93–1.31]	1.38 [1.13–1.62]	1.20 [0.96–1.43]
Not anxious (*N* = 87)	1.98 [1.83–2.14]	2.03 [1.91–2.15]	2.41 [2.32–2.49]
* p* value	< .001	< .001	< .001
Depressed (*N* = 39)	1.13 [0.93–1.33]	1.33 [1.13–1.53]	1.40 [1.18–1.61]
Not depressed (*N* = 81)	2.03 [1.88–2.18]	2.10 [1.98–2.23]	2.41 [2.31–2.51]
* p* value	< .001	< .001	< .001
Stressed (*N* = 57)	1.24 [1.07–1.41]	1.44 [1.27–1.62]	1.48 [1.31–1.65]
Not stressed (*N* = 65)	2.17 [2.02–2.32]	2.18 [2.05–2.31]	2.57 [2.48–2.65]
* p* value	< .001	< .001	< .001

N Number of patients

Young, < 60 years; Middle-age, 60–70 years; Elderly, > 70 years

^#^Middle-age versus elderly

Mean (95% confidence interval) comparisons with ANCOVAs (scores adjusted for age, sex, risk factors, and disease severity within diagnosis) and post hoc Bonferroni correction;

^*^HADS Hospital Anxiety and Depression Scale with cut-off score ≥ 8 for the anxiety/depression subscales and ≥ 12 for the general distress factor

Diagnosis (Table 2): Patients with MI reported significantly higher HeartQoL global scale and both physical and emotional subscale scores than patients with either AP or IHF.

MacNew (Table 6): Patients reporting higher MacNew global HRQL scores (top 33% of actual scores reported significantly higher HeartQoL global and both physical and emotional subscale scores than patients reporting lower MacNew global HRQL scores (bottom 33% of actual scores).

HADS anxiety (Table 6): Significantly higher HeartQoL global scale and both physical and emotional subscale scores were observed in patients who were not anxious than in patients who were anxious;

HADS depression (Table 6): Patients who were not depressed reported significantly higher HeartQoL global scale and both physical and emotional subscale scores than reported by patients who were depressed (*p* < 0.001);

HADS distress (Table 6): Significantly higher HeartQoL global scale, physical, and emotional subscale scores were observed in patients who were not distressed than in patients who were distressed;

Sex (Table 6): In the total group, female patients reported significantly higher HeartQoL global scale and both physical and emotional subscale scores than male patients; HeartQoL global scale and emotional subscale scores were significantly higher in females than males in patients with AP and in patients with IHF; in patients with MI, HeartQoL global scale and physical subscale scores were significantly higher in females than males.

Age (Table 6): The only significant HeartQoL score difference by age was a higher physical HeartQoL subscale score in middle-aged patients compared to elderly patients in the total group.

## Discussion

The Italian-language version of the 14-item HeartQoL questionnaire demonstrated adequate internal consistency, convergent, divergent and discriminative validity and therefore may be recommended for the use in clinical practice and research to assess and compare HRQL in Italian-speaking IHD patients with AP, MI or IHF. The psychometric properties of the Italian version of the HeartQoL were evaluated on a sample of 472 patients with either AP, MI or IHF from five Italian centres in North-East, North-West, Central, and Southern Italy and therefore largely representative of the demographic, social, cultural, and economic characteristics of the Italian population. Further, these psychometric properties are consistent with the original validation study in patients with AP, MI or IHF, [13] the English HeartQoL version based on patients with AP or MI in the USA, [30] the German HeartQoL version in patients with AP, MI or IHF, [14] The Iranian HeartQoL version in patients with MI, [31] the EuroAspire IV study in chronic coronary syndrome patients [32] and also with validation studies in patients with atrial fibrillation, [16] implantable cardioverter defibrillator recipients [17] or following valve surgery [18]. In addition, the original two-factor structure [12], determined with Mokken scale analysis [27], was confirmed with strong physical (Hi 0.67) and emotional (Hi 0.68) domains being comparable with the original [13] and more recent validation studies. [14, 16, 17, 30, 32].

With higher HRQL scores (range 0–3) indicating better HRQL, the mean HRQL scores in the Italian HeartQoL total group were 2.0 ± 0.07 on the global scale, 1.9 ± 0.08 on the physical subscale and 2.1 ± 0.08 on the emotional subscale and all scores reported by patients with MI indicated significantly better HRQL than reported by patients with either AP or IHF. With moderate floor and ceiling effects present when > 15% patients report the lowest score (= 0; floor) or the highest score (= 3; ceiling), [26] floor and ceiling effects on the global scale and physical subscale in the total group and each diagnosis were minimal indicating detection of both deterioration and improvement. However, with the emotional subscale, although all floor effects and ceiling effects in patients with AP or IHF were minimal, the ceiling effects were moderate in the total group and in patients with MI indicating limited detection of improvement. As this outcome is consistent with the original HeartQoL study [13] and other validation studies [14, 16–18, 30, 32] assessing improvement in HeartQoL emotional HRQL may be more challenging than either global or physical HRQL.

The Italian HeartQoL demonstrated high internal consistency reliability for each subscale, exceeding the recommended criterion of 0.70 for group HRQL comparisons with Cronbach’s α ≥ 0.92 on both the global scale and physical subscale and ≥ 0.84 on the emotional subscale in the total group and each of the three diagnoses. This is consistent with the previous validation studies [14, 16, 17, 30, 32] indicating that the items captured in the HeartQoL reflect the same constructs. Convergent validity refers to how closely scales measuring similar constructs are correlated, and was demonstrated across diagnostic groups with strong correlations (r > 0.70) between the physical and emotional subscales of HeartQoL and corresponding subscales of MacNew; with significantly lower correlations between dissimilar constructs, divergent validity was also confirmed.

Discriminative validity was determined using the ‘known groups’ approach [26] with the expectation that HRQL scores would distinguish between pre-identified groups. Of the total of 84 discriminative validity calculations in the total cohort and each diagnosis, 70 (83%) were fully confirmed; of the 14 calculations that were not significant, 11 (79%) were on the age and 3 (21%) on the sex variables. The discriminative validity of the Italian HeartQoL was fully confirmed for diagnosis as patients with MI reported significantly higher HeartQoL scores than patients with either AP or IHF which is largely consistent with both the original validation study [13] and the German validation study [14] as well as in the English HeartQoL where patients with MI reported higher HRQL scores than did patients with AP. [30] Discriminative validity was also fully confirmed with HADS anxiety/depression scales in the total group and in each diagnosis which is consistent for HADS anxiety and depression in the original study [13] and previously published HeartQoL studies. [14, 16–18, 30, 32] As reported in the German HeartQoL validation publication [14] the Italian HeartQoL HRQL scores were significantly higher in patients not reporting HADS distress compared to those reporting distress. Discriminative validity was largely confirmed for sex in this study with females reporting significantly better HRQL than males but this is not consistent with the EuroAspire IV study in chronic coronary syndrome patients [32], the German HeartQoL observations in patients with AP, MI or IHF [14] or the studies in Danish patients with atrial fibrillation [16] or following valve surgery [18] where males reported higher HRQL, although not always significant, than females. Finally, except for significantly higher physical HeartQoL scores in middle-aged patients than in elderly patients, discriminative validity was not confirmed for age in the present study. There was a trend for lower HRQL to be reported by elderly Italian patients which is largely consistent with the EuroAspire IV study [32] but not with the German HeartQoL observations [14] where the hypothesized lower HRQL scores in older patients was not consistently met.

## Limitations and strengths of the study

In every questionnaire with a relatively small number of items per scale, the amplitude of assessment of differences or changes in HRQL may be limited. Furthermore, examination of test–retest reproducibility was not possible with the cross-sectional design of the study which is also the major limitation of the present validation study not allowing for the determination of responsiveness. Future studies will be needed to determine the evaluative validity of the Italian HeartQoL questionnaire. The strength of the study lies in the demonstration of adequate key psychometric attributes of internal consistency reliability and convergent, divergent, and discriminative validity in Italian-speaking patients with ischemic heart disease.

## Conclusion

The Italian version of the HeartQoL questionnaire demonstrated satisfactory psychometric characteristics showing excellent internal consistency reliability and adequate convergent, divergent, and discriminative validity in Italian speaking patients with IHD and its three major diagnoses (AP, MI, and IHF). The Italian HeartQoL can be recommended to clinicians and researchers to estimate and compare the impact of IHD on patients’ HRQL.

## Abbreviations

AP: Angina pectoris
HRQL: Health-related quality of life
IHD: Ischemic heart disease
IHF: Ischemic heart failure
MI: Myocardial infarction
